# AI in Healthcare: Time-Series Forecasting Using Statistical, Neural, and Ensemble Architectures

**DOI:** 10.3389/fdata.2020.00004

**Published:** 2020-03-19

**Authors:** Shruti Kaushik, Abhinav Choudhury, Pankaj Kumar Sheron, Nataraj Dasgupta, Sayee Natarajan, Larry A. Pickett, Varun Dutt

**Affiliations:** ^1^Applied Cognitive Science Laboratory, Indian Institute of Technology Mandi, Mandi, India; ^2^RxDataScience, Inc., Durham, NC, United States

**Keywords:** time-series forecasting, persistence, autoregressive integrated moving average (ARIMA), multilayer perceptron (MLP), long short-term memory (LSTM), ensemble, medicine expenditures, neural networks

## Abstract

Both statistical and neural methods have been proposed in the literature to predict healthcare expenditures. However, less attention has been given to comparing predictions from both these methods as well as ensemble approaches in the healthcare domain. The primary objective of this paper was to evaluate different statistical, neural, and ensemble techniques in their ability to predict patients' weekly average expenditures on certain pain medications. Two statistical models, persistence (baseline) and autoregressive integrated moving average (ARIMA), a multilayer perceptron (MLP) model, a long short-term memory (LSTM) model, and an ensemble model combining predictions of the ARIMA, MLP, and LSTM models were calibrated to predict the expenditures on two different pain medications. In the MLP and LSTM models, we compared the influence of shuffling of training data and dropout of certain nodes in MLPs and nodes and recurrent connections in LSTMs in layers during training. Results revealed that the ensemble model outperformed the persistence, ARIMA, MLP, and LSTM models across both pain medications. In general, not shuffling the training data and adding the dropout helped the MLP models and shuffling the training data and not adding the dropout helped the LSTM models across both medications. We highlight the implications of using statistical, neural, and ensemble methods for time-series forecasting of outcomes in the healthcare domain.

## Introduction

Healthcare costs are rising, and patients need to manage their healthcare expenditures on medications (Bertsimas et al., 2008). Predicting medication cost in the future could help patients better manage patient-related healthcare expenditures (Zhao et al., 2001). To predict medication costs, one needs data concerning patients' medicine-purchase patterns. Currently, there exist significant amounts of digital healthcare data that can provide helpful insights into healthcare expenditures, and these data could bring about positive changes in healthcare policymaking (Farley et al., 2006). Although there exist data, accessing these data is a major challenge due to privacy concerns of patients, hospitals, insurance companies, and pharmaceutical companies. One way of overcoming this challenge is via anonymizing healthcare records and medicine information so that connections to specific individuals or entities are lost. Using anonymization, prior research has attempted time-series forecasting of different healthcare costs (Ash et al., 2000; Zhao et al., 2001, 2005; Farley et al., 2006). Here, most of the existing attempts have used statistical or machine-learning methods like regression (Ash et al., 2000; Zhao et al., 2001, 2005; Powers et al., 2005), classification trees (Robinson, 2008), and clustering (Bertsimas et al., 2008). Although prior research has performed the time-series forecasting in healthcare data, a major challenge is selection of the appropriate predictive model to use for performing analyses (there are very few suggestive forecasting algorithms for healthcare data due to the newness of this domain and its datasets). A way of overcoming the model selection challenge is by evaluating the predictions from existing forecasting methods with other recent methods in literature (Makridakis et al., 2018). For example, recently, certain recurrent machine-learning methods (e.g., long short-term models) have been proposed (Gamboa, 2017). These recurrent methods have been used for supervised learning of features for time-series forecasting (Gamboa, 2017; Miotto et al., 2017). These recurrent methods could provide improvements over existing statistical time-series techniques [e.g., autoregressive integrated moving average (ARIMA)], which are often dependent on the hand-crafted features requiring expert knowledge in the field.

A popular statistical time-series method is the ARIMA model (Newbold, 1983). The ARIMA model is popular because of its statistical properties (e.g., moving averages) to find its parameters (Box et al., 2015). ARIMA models generally use the historical values of a univariate time series to predict the time series' future values. A special case of the ARIMA model is a persistence model, where the future value in a time series is equal to its preceding value (thus, a persistence model has only one autoregressive term and no moving average terms). Given its simplicity, a persistence model could serve as a good baseline to compare other models. However, one challenge in persistence and ARIMA models is their pre-assumption of linearity in the underlying time series, which may be insufficient in various practical scenarios (e.g., healthcare datasets) that contain non-linear time-series data (Zhang, 2003).

To overcome the challenge of linear statistical time-series models, many non-linear machine-learning models like artificial neural networks (ANNs) have been proposed in the literature (Zhang, 2003, 2007; Kihoro et al., 2004; Kamruzzaman et al., 2006). ANNs belong to the data-driven approach, where training depends on the available data with a little prior rationalization regarding relationships between variables (Zhang, 2003). ANNs do not make any assumptions about the statistical distributions of the underlying time series and they can naturally perform non-linear modeling (Zhang, 2003). As a result, ANNs are self-adaptive by nature (Zhang et al., 1998; Zhang, 2003; Kamruzzaman et al., 2006).

Prior research has carried out comparative studies between ARIMA models and ANNs for performing time-series forecasting of stock-market data (Jain and Kumar, 2007; Adebiyi et al., 2014). This research showed that ANNs are a good alternative to the ARIMA approach, particularly in the case of non-linear time series and for long-term forecasting (Jain and Kumar, 2007; Adebiyi et al., 2014). There are different ANN-based forecasting models in the literature, where the most popular model is the multilayer perceptron (MLP) (Zhang et al., 2001). MLP models are memory-less, and they use the feed forward neural network architecture, which applies a supervised learning technique called back propagation algorithm for training the neural network (Koskela et al., 1996; Zhang, 2007).

Besides ARIMA and ANNs, newer models of time-series forecasting have been developed using recurrent memory-based approaches (Gamboa, 2017; Miotto et al., 2017). Among these models, memory-based recurrent neural network (RNN) architectures (Elman, 1990) and its variants, namely, the long short-term memory (LSTM) models (Hochreiter and Schmidhuber, 1997) and the gated recurrent unit (GRU) models (Chung et al., 2014), have been studied for extracting informative patterns from sequential healthcare data and in classifying data on the basis of diagnostic categories (Lipton et al., 2015; Che et al., 2018). There are several applications of RNN approaches in healthcare for performing predictions. For example, Lipton et al. (2015) have used LSTMs to perform diagnosis classification from clinical measurements of patients in pediatric intensive unit care. Pham et al. (2016) developed a dynamic memory model using LSTMs to predict future medical outcomes. Razavian et al. (2016) have used LSTM, RNN, and two convolutional neural network (CNN) models to predict disease onsets from longitudinal lab tests. Ma et al. (2017) have used bidirectional RNN architecture for predicting patients' future health information. Kaushik et al. (2017) have used a stacked LSTM for univariate time-series predictions of monthly expenditures of patients for medication.

Furthermore, another way of addressing the model selection challenge is by combining the predictions of various models or ensembling (Adhikari et al., 2015). Here, a number of ensembling mechanisms have been proposed (De Gooijer and Hyndman, 2006; Jose and Winkler, 2008; Adhikari et al., 2015). The standard approach of ensembling is simple averaging that assigns equal weights to all forecasting component models (De Gooijer and Hyndman, 2006; Jose and Winkler, 2008). However, the simple averaging method may be sensitive to outlier values and unreliable for skewed distributions (Freitas and Rodrigues, 2006; Adhikari et al., 2015). To correct for outliers, certain weighted combination approaches have also been proposed in the literature (Armstrong, 2001; Adhikari and Agrawal, 2014). One approach is the least-square regression (LSR) approach that attempts to find the optimal weights by minimizing the sum of squared errors (SSEs) (Adhikari and Agrawal, 2014). Another approach is the average of in-sample weights (AIW) scheme, where each weight is computed as the normalized inverse absolute forecasting error of an individual model, as has previously been proposed (Armstrong, 2001).

Another challenge for time-series forecasting in the healthcare domain is ensuring that models reduce their overfitting in the underlying data (i.e., to ensure that models do not perform well during training and poorly during testing). One way of reducing overfitting is by evaluating different approaches like dropouts and data shuffling as part of different time-series forecasting algorithms in the healthcare domain (Srivastava et al., 2014; Brownlee, 2016; Kang et al., 2017). Dropout is a regularization technique that helps reduce the problem of overfitting in neural networks by dropping the nodes in the networks (Srivastava et al., 2014). Similarly, recurrent dropout is another approach to handle overfitting that drops the recurrent connections in RNNs. Additionally, data shuffling is known to improve the general learning of model during its training, where effects of serial data presentation are reduced (Brownlee, 2016; Kang et al., 2017).

In this research, we address several of the above challenges by evaluating the performance of memory-less neural network models (e.g., MLP) with memory-based neural network models (e.g., LSTM) for performing time-series predictions of longitudinal healthcare data. Due to the popularity of the ARIMA model (Zhang, 2003; Box et al., 2015), we evaluate the performance of this model against both memory-less and memory-based approaches in anonymized healthcare data. Moreover, motivated by the use of ensemble architectures, we evaluate the potential of a weighted ensemble model (Adhikari and Agrawal, 2014), which combines the best predictions of the individual ARIMA, MLP, and LSTM models via a dynamic weight updation technique. Here, we compare the performance of the ensemble model as well as the individual models (ARIMA, MLP, and LSTM) with a persistence model, which serves as a baseline model.

Furthermore, to overcome the overfitting challenge, we evaluate the shuffling of time-series data with and without dropouts across different neural network models. Thus, we compare four different variations: shuffle with dropout, shuffle without dropout, no shuffle with dropout, and no shuffle without dropout. When shuffling is present (shuffle), smaller supervised sets (mini-batches) containing attributes corresponding to the chosen look-back period are created and shuffled across the time series during network training. Shuffling is done to change the order in which we present supervised sets to the models. It helps the model to avoid the conditions of local minima while training and thus it is helpful to reduce overfitting. However, when shuffle is not present (no shuffle), the mini-batches are created in the order they occur in data and inputted into the network without shuffling. When dropout is present, we discard certain number of nodes and recurrent connections (if present) from the hidden layers. As the adverse effects of node dropout in RNNs are known (Bayer et al., 2013), we tried both node and recurrent connection dropouts in the LSTM model. When dropout is absent, no nodes or recurrent connections (if present) are discarded during training in the neural network. Overall, introducing dropouts in neural network models may help reduce the overfitting as dropouts reduce the total number of weights to be trained in the model.

Overall, we expect the ensemble model to perform better in predicting time-series healthcare data compared to its individual constituting models as well as the baseline model. One likely reason for this expectation is because the ensemble model's prediction is highly dependent on the individual models' best predictions (the ensemble is expected to give more weight to those model predictions that are accurate compared to those that are less accurate). Furthermore, we expect the neural network models to perform better compared to statistical models because of the presence of non-linearities in the time-series data and the ability of neural networks to account for these non-linearities. Also, we expect shuffling the training samples and adding dropouts to help improve models' performance. The likely reason behind this expectation is that shuffling helps models avoid getting trapped in local minima during training due to an ordered presentation of training samples and dropouts help reduce overfitting.

This research is novel in several ways. First, this research addresses a number of challenges related to data anonymity, model selection, overfitting, and ensembling of individual models. In particular, this research compares statistical, neural, and ensembling machine-learning architectures and proposes a novel ensembling architecture that combines the best predictions of several component models. Second, this research is first of its kind on these data that consider existing and newer forecasting algorithms for predicting patient-related healthcare expenditures on medicines. Third, this research considers novel combinations of data shuffling and dropout mechanisms in a systematic way across a number of neural architectures. As the potential of the evaluated algorithms is still to be explored for predicting patient-related healthcare expenditures on medicines, this research is expected to provide suitable benchmarks for future research on time-series healthcare datasets concerning expenditures. Additionally, we have implemented the persistence algorithm to obtain the baseline performance of the time-series forecasting problem. The forecasting models developed are also likely to be useful to a number of stakeholders like patients, hospitals, pharmacies, and drug manufacturers.

In what follows, first, we explain the datasets, evaluation metrics, and experiment design. Then, we explain the working of different statistical, neural, and ensemble models that are proposed for predicting patients' expenditures on different pain medications. Furthermore, we present our experimental results, where we compare persistence, ARIMA, MLP, LSTM, and ensemble model predictions. We close the paper by discussing the implication of using different statistical, neural, and ensemble methods for predicting healthcare outcomes.

## Datasets

In this research, we selected two pain medications (named “A” and “B” as provided in the Supplementary Material) from the Truven MarketScan dataset for our analyses, where Truven provides real-world healthcare data to analyze patterns and cost (Danielson, 2014).^1^ The selected medications are among the top 10 most prescribed pain medications in the US (Scott, 2014). Data for both medications range between 2nd January 2011 and 15th April 2015 (1,565 days). Every day, on average, about 1,428 patients refilled medicine A, and about 550 patients refilled medicine B. For both medicines, we prepared a univariate time series containing the daily average expenditures by patients on these medications, respectively. The average expenditure per patient for medicine on a day *t* was defined as per the following equation:

(1) $$\begin{array}{r} {Daily\;Average\;Expenditure}_{t}=\;i_{t}/j_{t} \end{array}$$

where *i* is the total amount spent in a day *t* on the medicine across all patients and *j* is the total number of patients who refilled the medicine in day *t*. This daily average expenditure was used to compute the weekly average expenditure for both medicines, where the weekly average expenditure was used to evaluate model performance. For our analyses, across both pain medications, we used the dataset between 2nd January 2011 and 30th July 2014 (1,306 days) for model training and the dataset between 31st July 2014 and 15th April 2015 (259 days) for model testing. Moreover, standardization was required before training the models because our time-series data had input values with different scales (Patro and Sahu, 2015). Therefore, we standardized/re-scaled the time series for both medicines within range [−1, +1].

### Evaluation Metrics

All models (ARIMA, MLP, LSTM, and ensemble) were fit to data at a weekly level using the following metrics: root mean squared error (RMSE; error) and *R*-square (*R*^2^; trend) (Yilmaz et al., 2010). As weekly average expenditure predictions were of interest, the RMSE and *R*^2^ scores and visualizations for weekly average expenditures were computed in weekly blocks of 7 days. Thus, the daily average expenditures per patient were summed across 7 days in a block for both training and test datasets. This resulted in the weekly average expenditure across 186 blocks of training data and 37 blocks of test data. We used the training data to train different models to perform their parameter calibration. After finishing the training, we used the test data to verify the performance of trained models, where, during test, model parameters were kept at the values found during training.

**Figure 1:**
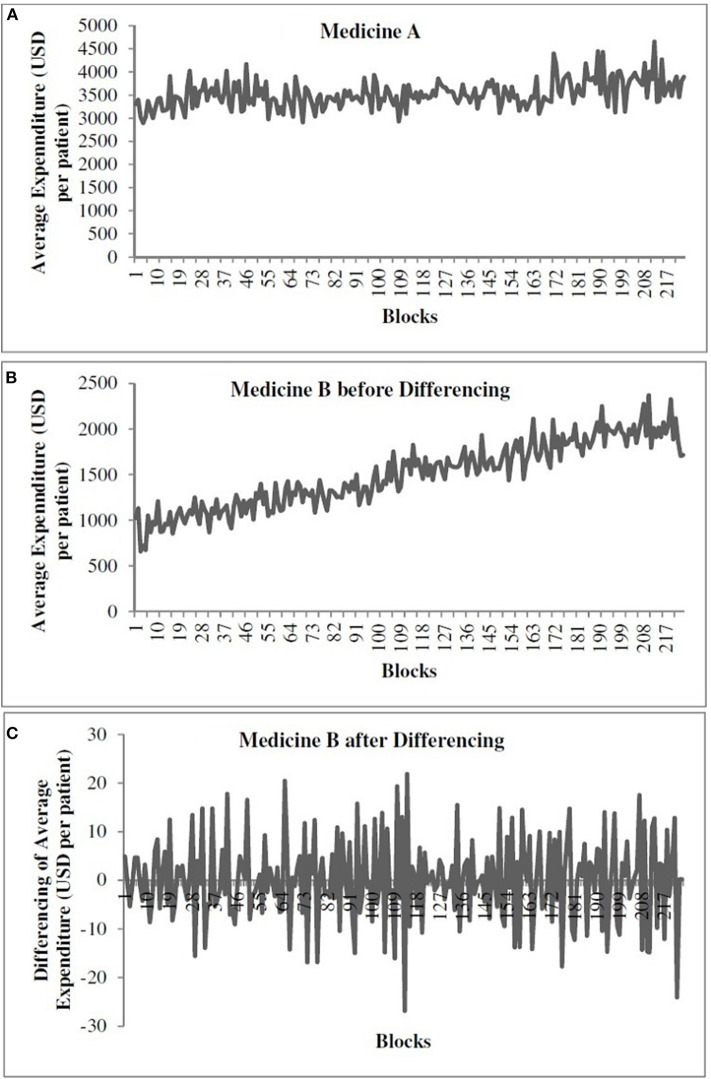
The weekly average expenditure (in USD per patient) for medicine A without differencing **(A)**, for medicine B before differencing **(B)**, and for medicine B after differencing **(C)**.

Figure 1A shows the time-series data for medicine A. Figures 1B,C show the time-series data for medicine B before and after differencing, respectively. The *x*-axis shows the weekly blocks, and the *y*-axis shows the weekly average expenditure (in USD per patient). In Figure 1, the first 186 blocks correspond to training data and the last 37 blocks correspond to the test data. We performed the augmented Dickey–Fuller (ADF) test (Dickey and Fuller, 1981) to determine the stationarity of a time series and confirm the value of *d* parameter. As shown in Figure 1A, the time series for medicine A was stationary (ADF statistics = −10.10, *p* < 0.05). However, as shown in Figure 1B, medicine B was non-stationary (ADF statistics = −2.20, *ns*). Thus, while training models for medicine B, we first made the time series stationary using first-order differencing (*d* = 1) (ADF statistics after one time differencing = −13.07, *p* < 0.05) (see Figure 1C). We used stationary data across both medicines to train the models. The predictions obtained from models for medicine B were first transformed to the non-stationary data before calculating the value of the objective function, i.e., RMSE and *R*^2^. We followed this procedure to train all the models for medicine B.

We calibrated all models to reduce error and capture trend in data. Thus, all models were calibrated using an objective function that was defined as the following: [RMSE/10 + (1–*R*^2^)]. The RMSE accounts for the error between the actual and predicted data. The smaller the RMSE, the smaller the error between model's predictions and the actual data. In addition, the *R*^2^ (between 0 and 1) accounts for whether the model's predictions follow the same trend as that present in the actual data. The larger the *R*^2^ (closer to 1), the larger the ability of the model to predict the trend in actual data. In our analyses, we found that the variation in RMSE term for both medicines was 10 times the variation in the 1–*R*^2^ term in the standardized data. Therefore, we divided the RMSE value by 10 to bring the RMSE value for both medicines in the range 0 to 1 (which is the range of 1–*R*^2^). In our parameter calibrations, the magnitude of the RMSE/10 term and the 1–*R*^2^ term was comparable across all models and medications. Hence, we used [RMSE/10 + (1–*R*^2^)] as the objective function because variation in both terms was similar and in [0, 1]. Overall, the objective function ensured that the obtained parameters minimized the error (RMSE) and maximized the trend (*R*^2^) on the weekly average expenditure per patient between model and actual data. Through model calibrations, different model parameters were varied across different ranges to find a set of parameters for which the model produced the least value of objective function on training data.

### Experiment Design

All models forecasted one-step ahead with walk-forward validation (Kaastra and Boyd, 1996). In one-step-ahead walk-forward validation, a model uses training data to make a prediction for the next time step. This prediction is then evaluated against the actual value. Next, the actual value corresponding to the prediction is added to the training data and the process is repeated by predicting the value for the next time step.

## Materials and Methods

### Models

In this section, we explain the working of different models like persistence (baseline), ARIMA, MLP, LSTM, and ensemble models.

#### Persistence

The persistence model uses the value at the previous time step to predict the value at the next time step. This model is implemented to obtain the baseline performance in the time-series forecasting problem.

#### Autoregressive Integrated Moving Average

In an ARIMA model (Newbold, 1983), the future value of a variable is assumed to be a linear function of several previous observations and random errors. An ARIMA model is defined as ARIMA (*p, d, q*):

*p*: order of the autoregressive part (AR);

*d*: degree of first differencing involved;

*q*: order of the moving average part (MA).

AR stands for “autoregressive,” and it is a stochastic process whose output values are linearly dependent on the weighted sum of its previous values and a white noise error (Newbold, 1983). MA stands for “moving average,” and it describes a stochastic process whose output value is linearly dependent on the weighted sum of a white noise error and the error term from previous periods (Newbold, 1983). One of the tasks for building the ARIMA model is to determine the value of (*p, d, q*). Classically, the autocorrelation and partial autocorrelation function plots have been used in the literature to determine the approximate range of *p* and *q* parameters, respectively (Newbold, 1983). Next, this range for *p* and *q* parameters can then be used in a grid search procedure (Whitley, 1994) to decide the precise values of these parameters. Following this methodology, we created the autocorrelation and partial autocorrelation function plots first and, then using these plots, we set the lower and upper bound values in the grid search for the *p* and *q* parameters.

The value of *d* indicates the number of times we need to difference the time series to make it stationary. We performed the ADF test (Dickey and Fuller, 1981) to determine the stationarity of a time series and find the value of the *d* parameter.

The underlying time series has the following form in the ARIMA model:

(2) $$y_{t}{}^{\prime }=c+\Phi_{1}y_{t-1}{}^{\prime }+\ldots +\;\Phi_{p}y_{p-1}{}^{\prime }+\theta_{1}e_{t-1}+\ldots +\theta_{q}e_{t-q}+e_{t}$$

$$\begin{array}{l} \text{AR}:\Phi_{1}y_{t-1}{}^{\prime }+\ldots +\;\Phi_{p}y_{p-1}{}^{\prime }\\ \text{MA}:\theta_{1}e_{t-1}+\ldots +\theta_{q}e_{t-q}+e_{t} \end{array}$$

where *c* is a constant, $y_{t}{}^{\prime }$ is the differenced series of observations (refer to Equation 2), *e*_*t*_ is the random error or white noise at a time period *t*, Φ_1_, Φ_2_, … Φ_*p*_ are the coefficients of the autoregressive part of the *p*th order, and θ_1_, θ_2_, … θ_*q*_ are the coefficients of the moving-average part of the *q*th order part, respectively.

#### Multilayer Perceptron

**Figure 2:**
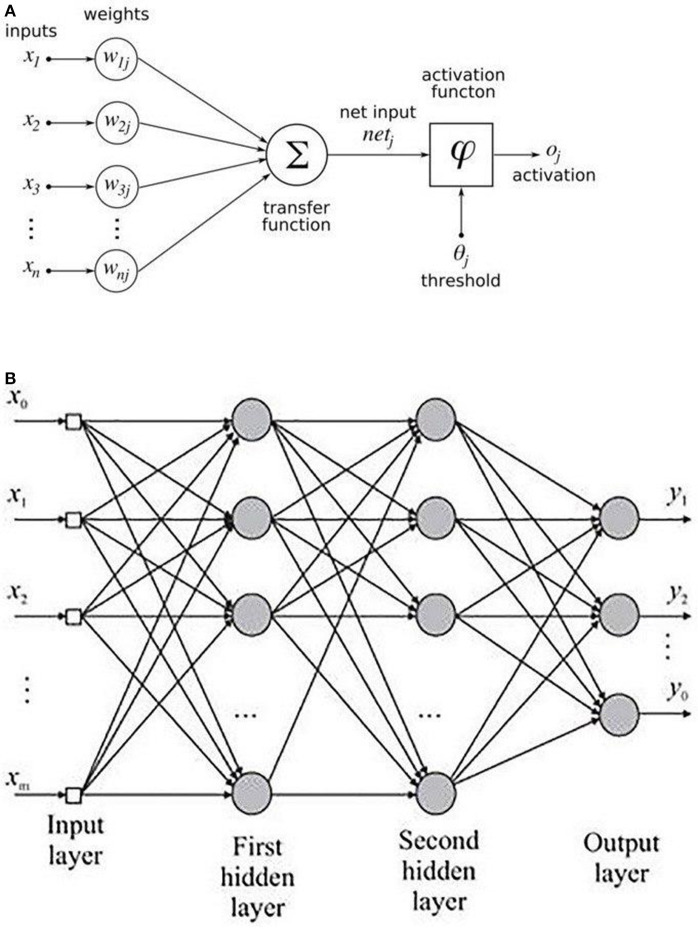
**(A)** Rosenblatt's perceptron and **(B)** Architectural graph of an MLP with two hidden layers.

An MLP is a variant of the original perceptron model (see Figure 2A) proposed by Rosenblatt (1961).

A neuron (represented as ∑ in Figure 2A) computes a weighted sum of the inputs, followed by a non-linear activation φ of the calculated sum, as shown in Equation 3. Neural network architectures are considered as the universal function approximators because of the presence of activation functions. An activation function helps to generate mappings from inputs to outputs, and it provides the neural network model the ability to learn complex data representations (Chung et al., 2016). There are a number of activation functions proposed in literature, which include the sigmoid, tanh, and ReLU (Krizhevsky et al., 2012; Chung et al., 2016). Krizhevsky et al. (2012) have shown that the sigmoid and tanh activation functions suffer from the problem of vanishing gradient, while the ReLU activation function overcomes the vanishing gradient problem and provides faster convergence. Also, the function is computationally efficient to compute (Krizhevsky et al., 2012). Thus, based on the literature, we used the ReLU activation function in the MLP model. The output *o*_*i*_ of a neuron in the MLP was defined as per the following equation:

(3) $$\begin{array}{r} o_{i}=\;\varphi \;\left(\overset{d}{\underset{j=1}{\sum }}\left(x_{j}w_{ij}+b_{j}\right)\right) \end{array}$$

where *d* is the length of the input vector, *x*_*i*_ is a single instance of the input vector, and *b*_*j*_ and *w*_*ij*_ are the bias and weights associated with each *x*_*j*_. Figure 2B shows the architecture of an MLP with two hidden layers. A typical MLP is composed of multiple hidden layers, with multiple neurons in each layer where every neuron in a layer (say *i*) is fully connected to every other neuron in the next layer (i.e., *i* + 1).

#### Long Short-Term Memory

**Figure 3:**
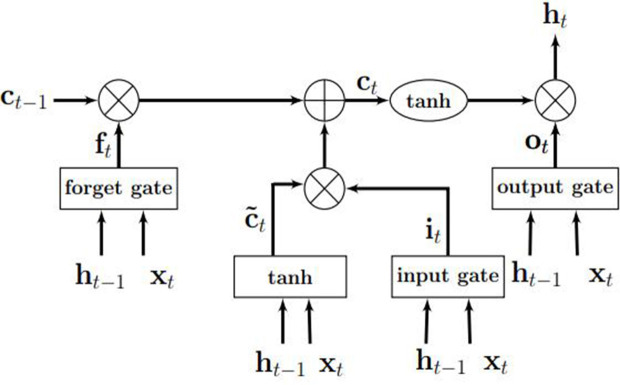
An illustration of an LSTM memory cell. Source: Gupta and Dinesh (2017).

An LSTM model is an RNN model with the capacity of remembering (i.e., memory) the values from earlier stages in the network. The architecture of an LSTM consists of units called memory cells. Figure 3 shows an LSTM memory cell containing self-connections and special multiplicative units called gates. These connections remember the temporal state of the memory cells and the gates control the flow of information. Each memory cell contains an input gate, an output gate, and a forget gate. The flow of input activations is controlled by the input gate, while the flow of cell activations into the remaining network is controlled by the output gate. Furthermore, the internal state of the cell is scaled by the forget gate and is added back to the cell as input through a self-recurrent connection. In Figure 3, *c*_*t*−1_ is the previous cell state, *h*_*t*−1_ is the previous cell output, *x*_*t*_ represents input to the memory cell, *c*_*t*_ represents the new cell state, and *h*_*t*_ represents the output of the hidden layer at time *t*.

#### Ensemble

In this research, we propose a novel ensemble architecture that combines the best predictions from multiple models to predict outcomes of interest. The ensembling is expected to reduce the bias and variance in the individual prediction models by using conventional ensembling techniques like mode or average (De Gooijer and Hyndman, 2006). In the case of a mode or average ensembles, the most frequently occurring or the average prediction from multiple models are used as the final prediction from the ensemble (De Gooijer and Hyndman, 2006). To overcome the challenges of the mode or average ensembling techniques, the ensembling approach in this research uses a weighted average ensemble, where different individual model's best predictions are dynamically weighted (Adhikari and Agrawal, 2014). In our ensemble model, the weights associated with different best model predictions from ARIMA, MLP, and LSTM models are calibrated using a normalized exponential weighting algorithm (Adhikari et al., 2015). More details about this model's calibration are presented ahead in this paper.

### Models Training

#### Time-Series Forecasting Using ARIMA

As explained above, the ARIMA model possessed three parameters *p* (order of autoregressive part), *d* (degree of differencing), and *q* (order of moving average part) (Newbold, 1983). The *p* term describes the previous time steps of a time series used for predicting the future value. The *q* term describes the previous error terms used to predict the future value (Box et al., 2015). The autocorrelation and partial autocorrelation function plots revealed the best range for the variation of *q* and *p* to be integer values between [0, 5]. Thus, the grid search was applied by passing the integer values in the range [0, 5] for both *p* and *q* to decide the precise values of these parameters. Through grid search, we checked all the integer combinations of *p* and *q* between 0 and 5 and chose the values for which we obtained the least value of our objective function. The parameter values for which we obtained the least value of the objective function were termed as the optimum values of these parameters.

The value of *d* indicates the number of times we need to difference the time series to make it stationary. Based on the ADF test (Dickey and Fuller, 1981), we found the time series of medicine A to be already stationary (*d* = 0). However, the time series for medicine B was non-stationary and required one-time differencing (*d* = 1) (the ADF test confirmed that the one-time differenced time series for medicine B was stationary). As medicine A was stationary (see Figure 1A), the lag value was calculated on the original time series. In the ARIMA model, we obtained lag value of 2 time steps for medicine A. However, in case of medicine B (non-stationary time series; Figure 1B), the lag value was calculated after one-time differencing. Thus, on the differenced series (Figure 1C), we obtained a lag value of 2 time steps for medicine B [however, on the original (non-differenced) series, it was a lag of 3 time steps for medicine B]. The time series for each medication was transformed into a supervised learning problem before model training. For medicine A, we used the two previous time steps (*t* – 2) and (*t* – 1) of the original series as inputs to a model and the current time step (*t*) as the output from the model. For medicine B, we trained the models using the differenced series where the models utilized past 3 time steps (*t* – 3), (*t* – 2), and (*t* – 1) of the original series (i.e., lag = 2 for the differenced series) as inputs to a model and the current time step (*t*) as the output from the model.

#### Time-Series Forecasting Using MLP and LSTM

Both MLP and LSTM models were trained across each of the two medications on the training dataset. On medicine A, MLP and LSTM models were trained on original time series. On medicine B, MLP and LSTM models were trained on the differenced time series. We used the genetic algorithm (Whitley, 1994) with 20% mutation and 80% crossover rate to tune the following hyper-parameters using training data: number of hidden layers, number of neurons in a layer, batch size, and epochs. The hyper-parameters were varied in the following ranges: hidden layers (1, 2, 3, and 4), number of neurons in a layer (4, 8, 16, 32, and 64), batch size (5, 10, 15, and 20), and number of epochs (8, 16, 32, 64, 128, 256, and 512). First, we hand-tuned the abovementioned parameters in MLP and LSTM models to obtain an idea about the minimum and maximum value of parameter ranges. The minimum and maximum values of a hyper-parameter were the ones where we did not find improvements in model's fit by choosing a value lower than the minimum and higher than the maximum. Next, we varied the hyper-parameters between the minimum and maximum and we used genetic algorithm to tune the hyper-parameters (layers, neurons, batch size, and epochs) of the MLP and LSTM architectures. The genetic algorithm used RMSE/10 + (1–*R*^2^) as the objective function, and it was run for 100+ generations. We stopped the genetic algorithm when the value of objective function did not change for the past 20 generations. Out of these generations, we obtained the set of hyper-parameters for which we got the minimum value of the objective function (we call these optimum parameters). After training, all the models were validated on test datasets. During training, we did not want to give advantage to the memory-based LSTM model over the memory-less MLP model. Therefore, we varied the same range of hyper-parameters for both the MLP and LSTM models. The LSTM model is a memory-based model that could benefit by keeping larger lag values. However, we did not find improvements in RMSEs in the LSTM model for different lag values between 2 and 8. To ensure that the lag was kept the same across all models, we chose a lag value of 2 time steps across MLP and LSTM models for medicine A (using original series). For medicine B, we trained the MLP and LSTM models using the differenced series where the models utilized past 2 time steps of the differenced series (i.e., lag = 3 of the original series). We used these models to predict patients' daily average expenditure on medicines. After getting the predictions for 259 days (test data), we summed these daily average expenditures on blocks of 7 days to get the weekly average expenditures by patients on medicines.

In addition, in each MLP and LSTM model, we varied shuffling of training data and dropout of nodes as per the following combinations: shuffle with dropout, shuffle without dropout, no shuffle with dropout, and no shuffle without dropout. First, we converted the training time series for each medicine into smaller supervised sets (mini-batches) containing attributes corresponding to the chosen look-back period. When shuffling was present, the mini-batches were shuffled randomly across time series for each medicine. When shuffling was absent, we did not shuffle the mini-batches and presented these batches in sequential order to the neural network. Dropout is a regularization technique, which is used to tackle overfitting in neural networks (Srivastava et al., 2014). As per Srivastava et al. (2014), dropout can be implemented on any or all hidden layers in the network except the output layer. Based on this literature, when dropout was present, 20% of the hidden-layer nodes in the MLP model and 20% of the nodes or recurrent connections in LSTMs were randomly dropped out during model training. When dropout was absent, no nodes in the MLP model and no nodes or recurrent connections in LSTMs were removed from the hidden layers in the network.

Using the genetic algorithm, we obtained the best set of hyper-parameters for each medication for different combinations of dropouts and shuffling (the best set of hyper-parameters minimized the objective function) on training data. Next, we ran these best hyper-parameters 30 times for each of the four dropout and shuffle combinations (the model was run 30 times as there is a run-to-run variability in model's output) on training data. Among the 30 model runs, the run with the least objective function value was treated as the final prediction from the model for a dropout and shuffle combination. These final predictions with the least objective function value were then used along with the other models' final predictions to generate the predictions for the ensemble approach. However, all the models were finally compared based on their average performance across 30 runs. We used the ReLU activation function while training the MLP and LSTM models (Chung et al., 2016). These models were created in Keras v2.2.2 using Tensorflow backend v1.10.0 (Chollet, 2015). We used the same procedure for training the MLP and LSTM models for both the pain medications.

#### Time-Series Forecasting Using Ensemble

In order to train the ensemble model, we used the best predictions from ARIMA, MLP, and LSTM models on training data. As mentioned above, all these models were run 30 times. The best predictions from one of these 30 runs (the one with the lowest RMSE) in each model were used to generate the predictions from the ensemble model. The ARIMA model, however, is different from the neural network models as it gives the same predictions across the 30 runs. We used the predicted training data points (1–1,306 points) from the best run of the ARIMA, MLP, and LSTM models to compute the weights of the ensemble model. In order to calibrate the weights of ensemble model, we used the normalized exponential weighted algorithm (Adhikari et al., 2015).

##### Normalized exponential weighted algorithm

The working of this algorithm is presented in the box below. Given a set of *N* predictions from different models on the training data (line 1), this algorithm starts with assigning equal weight to all predictions (i.e., $\frac{1}{3}$ weight to the ARIMA, MLP, and LSTM model predictions; line 2). The assumption of each weight to be 1/*N* ensures that the sum of all model weights equal 1 at each step of the model training. Then, for each of the training samples (from 1 to *T*, where *T* = 1,306 in our data), the squared error between each model's prediction and actual data is computed (line 4). After each training sample, each model's weights are updated using the squared error for different model predictions (line 5), where the parameter η is a free learning-rate parameter. Finally, we normalize the weights obtained for each model's predictions by dividing them with the total sum of the weights across all models (line 6). We normalize these weights to bring all weights between 0 and 1. This process continues until all the 1,306 training samples are covered. To obtain the optimum weights corresponding to each model, we need to try different values of the η parameter. In this algorithm, we calibrated the value of the η parameter by varying its values from 0.0 to 1.0 in steps of 0.1. Therefore, we tried 11 different values of η parameter and obtained different weights for each model. We selected those weights for which we obtained the minimum value of objective function (i.e., minimum RMSE and maximum *R*^2^). We call these weights optimum weights for ARIMA, MLP, and LSTM models.

**Algorithm 1:** Normalized Exponential Weighted Algorithm

1: **Input:** $A\;model\;i\;predicting\;outcome\;f_{i}^{t}\;for\;round\;t\;and\;a$ *free parameter η*
2: $w_{i}^{1}\leftarrow \frac{1}{N}$ i = 1, …, N models (set initial weights of each model's predictions to 1/N such that sum of the weights is equal to 1
3: For training samples t = 1 to T and for each model i = 1 to N do
4: $l\left(f_{i}^{t}y_{t}\right)={\left(f_{i}^{t}-y_{t}{}^{2}\right)}^{2}$ (calculation of squared error where *y*_*t*_ is the actual value)
5: $w_{i}^{t+1}\leftarrow w_{i}^{t}e^{\eta l\left(f_{i}^{t},y_{t}\right)}$
6: $w_{i}^{t+1}\;\leftarrow w_{i}^{t+1}/\overset{N}{\underset{i=1}{\sum }}w_{i}^{t+1}$ (normalization of weights)

## Experimental Results

### Persistence Model

**Figure 4:**
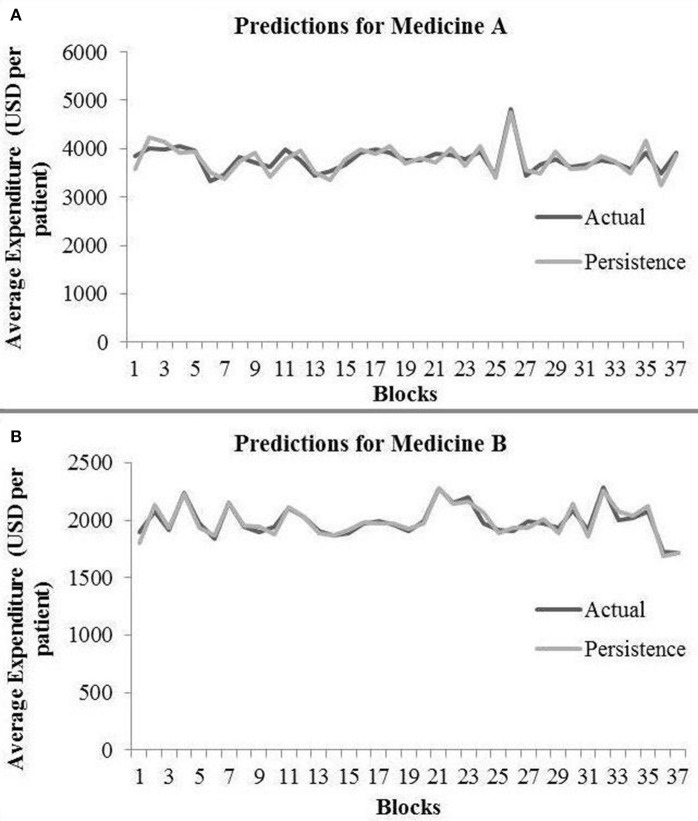
Average expenditure (in USD per patient) from the persistence model for medicine A **(A)** and for medicine B **(B)** in test data.

In the persistence model, we obtained RMSE = 145.68 (medicine A) and RMSE = 40.33 (medicine B) on test data. Similarly, we obtained *R*^2^ = 0.76 (medicine A) and *R*^2^ = 0.91 (medicine B) on test data. Figure 4 shows the persistence model's performance for medicine A (Figure 4A) and medicine B (Figure 4B) on the average expenditure per patient in test data.

### ARIMA Model

**Figure 5:**
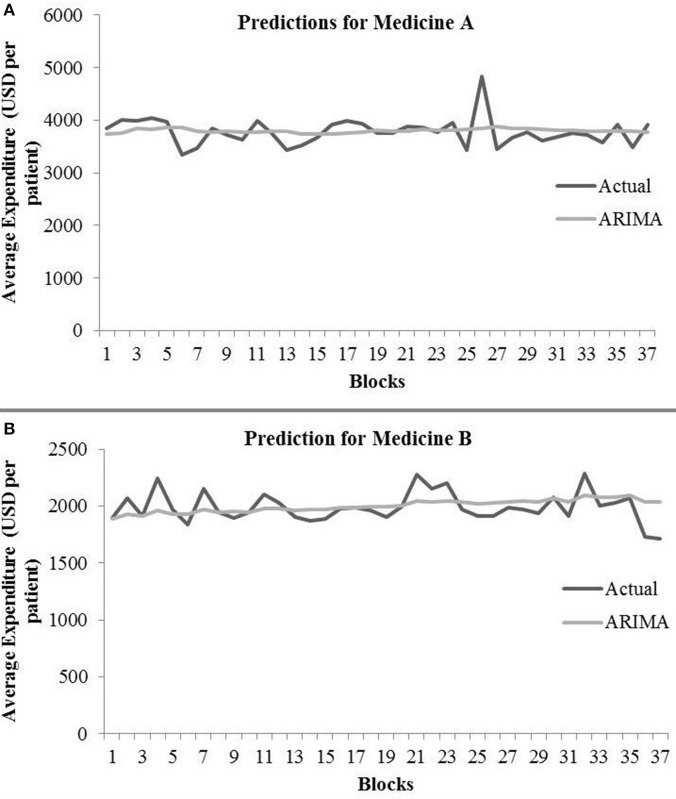
Average expenditure (in USD per patient) from the ARIMA model for medicine A **(A)** and for medicine B **(B)** in test data.

The ARIMA model for medicine A possessed the following parameters: *p* = 2, *d* = 0, and *q* = 1. The ARIMA model for medicine B possessed the following parameters: *p* = 2, *d* = 1, and *q* = 2. Table 1 shows the ARIMA model's RMSE and *R*^2^ on training and test data for both medicines. Figure 5 shows the ARIMA model's performance for medicine A (Figure 5A) and medicine B (Figure 5B) on the average expenditure per patient in test data. As seen in Figure 5 and Table 1, the model fits were poor across both training and test data for both medicines.

**Table 1:** ARIMA results during training and test.

**Medicine name**	**Train RMSE**	**Train *R*^**2**^**	**Test RMSE**	**Test *R*^**2**^**
A	248.72	0.14	260.97	0.0004
B	102.02	0.89	126.18	0.08

### MLP Model

**Figure 6:**
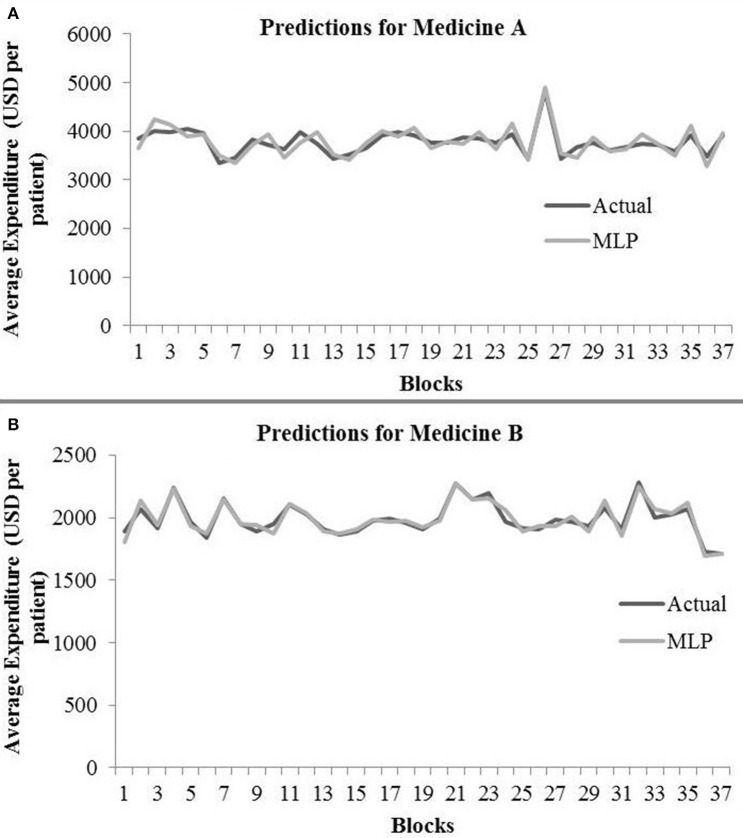
Average expenditure (in USD per patient) from the MLP model for medicine A **(A)** and for medicine B **(B)** in test data.

Table 2 shows the MLP model's RMSE and *R*^2^ on training and test data for different shuffle and dropout combinations on medicines A and B (shuffle with dropout, shuffle without dropout, no shuffle with dropout, and no shuffle without dropout). As shown in Table 2, the best RMSE (= 142.69) on test data was obtained for the no shuffle with dropout combination for medicine A, and this model contained 2 hidden layers, 4 neurons in each hidden layer, 15 batch size, and 16 epochs. On medicine B, we obtained the best RMSE (= 40.33) on test data for no shuffle and dropout combination. The corresponding MLP model for medicine B possessed 3 layers, 4 neurons in each layer, 5 batch size, and 128 epochs. Figure 6 shows the MLP model fits for medicine A (Figure 6A) and medicine B (Figure 6B) in test data for the best-performing model combinations. As shown in Figure 6, the MLP model fits were reasonably accurate for both medicines.

**Table 2:** MLP results during training and test.

**Medicine Name**	**Combinations of shuffle and dropout**	**Train RMSE**	**Train *R*^**2**^**	**Test RMSE**	**Test *R*^**2**^**
A	Shuffle with dropout	118.87	0.83	148.09	0.77
	Shuffle without dropout	134.10	0.79	153.80	0.74
	**No shuffle with dropout**	130.63	0.79	142.69	0.74
	No shuffle without dropout	136.59	0.77	154.45	0.74
B	Shuffle with dropout	50.59	0.97	49.03	0.88
	Shuffle without dropout	53.58	0.97	55.00	0.85
	**No shuffle with dropout**	43.35	0.98	40.33	0.91
	No shuffle without dropout	56.86	0.97	54.80	0.85

*The bold text highlights the variation with the lowest RMSE on test data*.

### LSTM Model

**Figure 7:**
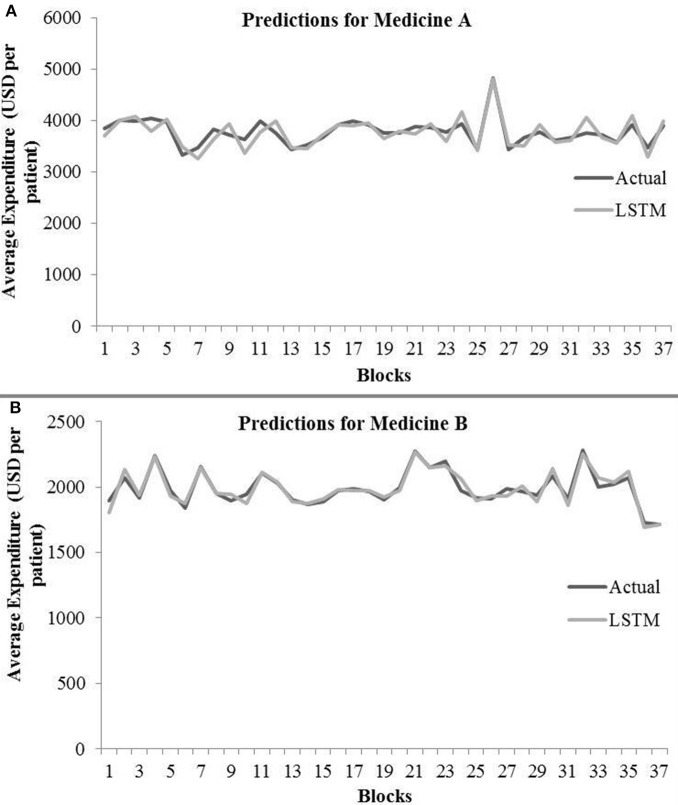
Average expenditure (in USD per patient) from the LSTM model for medicine A **(A)** and for medicine B **(B)** in test data.

Table 3 shows the LSTM model's RMSE and *R*^2^ on training and test data for different shuffle and dropout combinations on medicines A and B (shuffle with node and recurrent connection dropout, shuffle without dropout, no shuffle with node and recurrent connection dropout, and no shuffle without dropout). As shown in Table 3, the best RMSE (= 143.69) on test data was obtained for the shuffle and no dropout combination for medicine A, and this model contained 1 hidden layer, 4 neurons per hidden layer, 20 batch size, and 8 epochs. On medicine B, we obtained the best RMSE (= 40.30) on test data for shuffle and no dropout combination. The corresponding LSTM model possessed 1 layer, 4 neurons, 20 batch size, and 8 epochs. Figure 7 shows the LSTM model fits for medicine A (Figure 7A) and medicine B (Figure 7B) in test data for the best-performing model combinations. As shown in Figure 7, the LSTM model fits were reasonably accurate for both medicines.

**Table 3:** LSTM results during training and test.

**Medicine name**	**Combinations of shuffle and dropout**	**Train RMSE**	**Train *R*^**2**^**	**Test RMSE**	**Test *R*^**2**^**
A	Shuffle with node dropout	120.124	0.82	145.961	0.77
	Shuffle with recurrent connection dropout	130.33	0.78	155.29	0.72
	**Shuffle without dropout**	109.96	0.87	143.69	0.77
	No shuffle with node dropout	104.78	0.87	145.69	0.76
	No shuffle with recurrent connection dropout	129.35	0.82	200.42	0.67
	No shuffle without dropout	139.11	0.77	150.51	0.76
B	Shuffle with node dropout	43.36	0.98	40.34	0.91
	Shuffle with recurrent connection dropout	52.43	0.97	52.02	0.85
	**Shuffle without dropout**	43.35	0.98	40.30	0.91
	No shuffle with node dropout	43.33	0.98	40.35	0.91
	No shuffle with recurrent connection dropout	61.04	0.96	65.42	0.76
	No shuffle without dropout	45.29	0.98	43.42	0.91

*The bold text highlights the variation with the lowest RMSE on test data*.

### Ensemble Model

**Figure 8:**
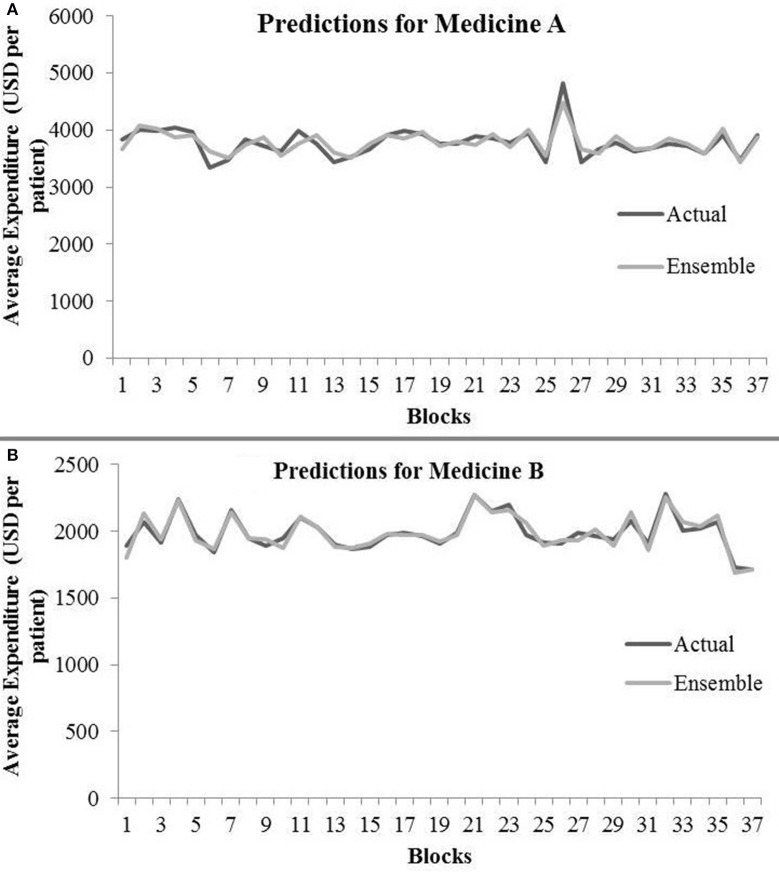
Average expenditure (in USD per patient) from ensemble model for medicine A **(A)** and for medicine B **(B)** in test data.

Table 4 shows the ensemble model's RMSE and *R*^2^ on training and test data on both the medicines. These results were obtained using the best predictions out of 30 runs from each of the ARIMA, MLP, and LSTM models. The ensemble results on medicine A were obtained with the following weights: 0.333 for ARIMA, 0.329 for MLP, and 0.329 for LSTM, with η = 0.2 as the learning rate parameter from the normalized exponential weighted algorithm. On medicine B, the ensemble results were obtained with the following weights: 0.000 for ARIMA, 0.231 for MLP, and 0.768 for LSTM. The ensemble model weights for medicine B were obtained for η = 0.9 using the normalized exponential weighted algorithm. Figure 8 shows the model fits from the ensemble model for medicine A (Figure 8A) and medicine B (Figure 8B). As shown in Figure 8, the ensemble model's fits (RMSE and *R*^2^) on test data were reasonably better compared to its individual constituting models.

**Table 4:** Ensemble model results during training and test.

**Ensemble model for medicines**	**Train RMSE**	**Train *R*^**2**^**	**Test RMSE**	**Test *R*^**2**^**
A	131.60	0.78	127.20	0.77
B	66.48	0.95	40.24	0.91

### Model Comparison

Table 5 shows the best test RMSE and best test *R*^2^ obtained over 30 runs from all the models for both the medicines. The average RMSE and *R*^2^ were obtained by averaging the RMSE and *R*^2^ across both medicines A and B. As seen in the table, both the MLP and LSTM models outperformed the persistence (baseline) as well as the ARIMA model. Also, the ensemble model performed the best among all the models for both the medicines on the test data. The overall trend across persistence, LSTM, and MLP models was similar and better compared to the ARIMA model.

**Table 5:** Best results over 30 runs on test data from different models across both medicines.

**Model**	**RMSE (A)**	**RMSE (B)**	***R*^**2**^ (A)**	***R*^**2**^ (B)**	**Average RMSE**	**Average *R*^**2**^**
Persistence	145.68	40.33	0.76	0.91	93.00	0.84
ARIMA	260.97	126.18	0.0004	0.08	193.58	0.04
MLP	142.69	40.33	0.74	0.91	91.51	0.83
LSTM	143.69	40.30	0.77	0.91	91.99	0.84
Ensemble	**127.20**	**40.24**	**0.77**	**0.91**	**83.72**	**0.84**

*The bold text highlights the model with the lowest RMSE on test data*.

## Discussions and Conclusions

The primary objective of this research was to compare the performance of existing statistical (persistence and ARIMA) and neural (MLP and LSTM) models with a novel ensemble model for forecasting patient-related expenditures on medications. Another objective of this paper was to systematically evaluate the advantages of shuffling of training data and adding of dropouts while training the neural network models individually or as a part of the ensemble model. Overall, we expected the ensemble model to perform better compared to the statistical and neural models. Also, we expected shuffling and dropouts to help models improve their performance. Overall, our results have met our expectations.

First, as per our expectation, the best performance in terms of error was found from the ensemble model, followed by MLP, LSTM, persistence, and ARIMA models. A likely reason for these results is that the persistence and ARIMA models are perhaps not able to capture the non-linearities present in the time-series data. Thus, overall, these models tended to perform not as well compared to other models. Also, overall, the neural network models (MLP and LSTM) performed similarly and better than the persistence and ARIMA models. That is likely because data for both medicines were non-linear and neural network models, by their design, could account for the non-linear trends in datasets. However, another reason for this result could be simply because the neural network models possess several weights (parameters), whereas the ARIMA model possesses only three parameters. These reasons for the MLP and LSTM models and the fact that increasing the lag in the LSTM model did not help this model improve its performance could also help explain the similarity in performance for the MLP and LSTM models. Furthermore, the novel ensemble model performed better compared to all the individual models because this model gave more weight to the accurate model predictions than the less accurate ones as well as dynamically adjusted its weights.

In our results, the ensemble model outperformed other statistical and neural models. This result may not be in agreement with prior literature that shows that ensembling approaches may only produce minor improvements (Hinton et al., 2015). A likely reason behind achieving substantial boost in performance through our novel ensemble model is the design of its training procedures. After getting the best model configurations (hyper-parameters) from individual models, we trained all individual models several times to obtain the best value of their objective function. Additionally, MLP and LSTM were trained with shuffle-and-dropout variations. Out of the several individual model runs, the ensemble model chose the best predictions to train its weight and learning-rate parameters. For calibrating the weights, the ensemble model dynamically adjusted its weights using a normalized exponential algorithm.

Third, we found that, in general, the MLP model performed better on test data with no shuffle and dropout combination across both medicines. A likely reason for this result could be that adding the dropout helped the MLP to generalize better with reduced overfitting (performance of MLP during test was comparable or better compared to that during training). In addition, MLP models do not possess memory to account for past presentations of training data. Thus, in the absence of memory, the sequential (non-shuffled) presentation of training data most likely helped the MLP models to improve their performance.

Fourth, for LSTM models, the best results on test data were obtained when input data were shuffled and dropouts were absent in the network. A likely reason for this result could be that the LSTM models contain memory, so they could save the past presentation of training data when shuffled mini-batches were passed to the network. Dropout did not reduce the overfitting in the LSTM model for medicine A. Furthermore, the results of medicine B were much better compared to those of medicine A. In order to reduce overfitting in the recurrent network (i.e., LSTM), we also implemented recurrent dropout that drops the recurrent connections in the network. With recurrent dropout, the lowest RMSE obtained was 155.29 on test data for medicine A. However, with the best shuffle-and-dropout condition, we obtained 143.69 RMSE for medicine A. Similarly, on medicine B, the RMSE increased from 40.30 to 52.02 by implementing recurrent dropout. Thus, the overall utility of dropout (to reduce overfitting) across the two medications was not found in this model. Overall, from our findings, we may conclude that shuffling the input training data may help the LSTM models and adding the dropout may help the MLP-based time-series forecasting models to perform better. Interestingly, the results of ensemble model showed no overfitting for both medicines. Thus, in contrast to using dropouts, the ensemble model's use may help reduce overfitting in data.

Beyond dropouts, prior research has proposed other regularization techniques such as L1 and L2 regularization for the problem of overfitting (Srivastava et al., 2014). These regularization techniques add a regularization term to the cost function to penalize the model for having several parameters. The parameter reduction leads to simpler models that likely reduce overfitting. In the future, we plan to apply the L1 and L2 regularization to evaluate their ability to reduce overfitting in data.

Additionally, the persistence (baseline) model performed better compared to the ARIMA model. A likely reason for this result could be the nature of datasets, where the average per-patient expenditure changed little between two adjacent blocks. Perhaps, due to these smaller changes in the expenditure, the persistence model was able to gain advantage over the ARIMA model. In fact, a smaller value of *p* and *q* parameters in the ARIMA model agreed well with the plausibility of the persistence model performing better across both medications.

This research work has a number of implications for healthcare data analytics. First, an implication from our results is that neural network models could be used in healthcare datasets to predict medicine expenditures. However, an ensemble of these neural models may improve the overall results further. Although we considered the weekly expenditure per patient on medications in this paper, our results are likely to hold for other patient-related healthcare expenditures. However, it may be prudent to test these models on other per-patient healthcare expenditures before using them in a production environment. Second, another implication of our results is that it may be expensive to train neural network and ensemble models; however, once these models are trained, they are easy and computationally and temporarily inexpensive to apply on new data.

Therefore, we believe that the neural and ensemble approaches would be useful to caregivers, patients, pharmacies, and pharmaceutical companies. For example, the proposed ensemble and neural models could be bundled into a mobile or desktop application that helps patients better manage their spending on medicine purchases by forecasting their future spending. Third, the proposed models may help pharmaceutical companies optimize their manufacturing processes and determine an attractive pricing for their medications. Furthermore, the proposed models could also be beneficial for medicine inventory management and pricing across pharmacies and hospitals.

Although the proposed ensemble approach performs relatively better compared to other statistical and neural approaches and is reproducible, it may require model retraining at regular time intervals especially when newer data are generated over time. Also, we believe that our results replicated on two pain medications show a certain amount of generalizability. However, it may be advisable to test the proposed ensembling approach against other types of medications with similar or dissimilar distribution to the current dataset as part of future research.

There are several possibilities for future research that could extend this work. For example, beyond predicting per-patient medicine expenditures, future research could use CNN models and other RNN models to predict disease risk and diagnose patient symptoms (Lipton et al., 2015; Maxwell et al., 2017). Here, RNNs like the GRUs (Chung et al., 2014) could be used and compared to regression-based techniques, i.e., support vector regression (Morid et al., 2017), to predict healthcare outcomes. Also, as the focus of this paper was on the applicability of statistical, neural, and ensemble architectures on longitudinal healthcare data, one could extend this comparison to other datasets with different challenges such as variability in data velocity, data missingness, and data types. In addition to weighted ensembles, there exists bagging, or bootstrap aggregating, which makes decisions based on the aggregated results of the sampled decision trees. Along with the weighted ensembles, one may use bootstrapped samples of the training data, thereby creating a random forest of models to create ensemble models. Still another possibility for future research is to extend univariate time-series forecasting to multi-variate time-series forecasting (Kaushik et al., 2019), where one uses other patient-related variables (both continuous and discrete) alongside per-patient expenditures on different medications. Some of these ideas form the immediate next steps in our research program on time-series forecasting of healthcare datasets.

## Data Availability Statement

All datasets generated for this study are included in the article/Supplementary Material.

## Author Contributions

SK and VD contributed to the conception and design of the study. SK wrote the first draft of the manuscript. SK and VD improved the final version of the manuscript draft. ND, SN, and LP helped in getting the data used in this paper. SK and AC organized the database and trained ARIMA, LSTM, and ensemble models. PS trained the MLP model. All authors contributed to manuscript revision, read and approved the submitted version.

### Conflict of Interest

ND, SN, and LP are employed by RxDataScience, Inc., USA. The remaining authors declare that the research was conducted in the absence of any commercial or financial relationships that could be construed as a potential conflict of interest.

## Footnotes

^1^Due to a non-disclosure agreement, the actual names of the two pain medications have not been disclosed. Also, by anonymizing medicine names, we ensure that this research and its outcomes do not target specific drugs, drug manufacturers, hospitals, pharmacies, or patients in the real world.
